# Gastric cancer presenting with solitary gigantic pelvic metastasis

**DOI:** 10.7555/JBR.26.20110056

**Published:** 2012-04-15

**Authors:** Qi Zheng, Kejun Nan, Yu Yao

**Affiliations:** Department of Medical Oncology, the First Affiliated Hospital, Xi'an Jiaotong University, Xi'an, Shaanxi 710061, China.

**Keywords:** gastric cancer, bone metastasis, pelvic

## Abstract

Bone metastasis of gastric cancer is relatively uncommon in clinical practice. Moreover, it is all the more unusual for the primary presentation of gastric malignancy to be bone metastasis. Here, we describe a male patient who complained of pain and edema in his right lower extremity. Further assessment by computed tomography and positron emission tomography revealed an abnormally thickened gastric cardia and a giant neoplasm in the right pelvis with bone damage. Consequently, the finding of adenocarcinoma cells in pelvic and cardia biopsy specimens contributed to the diagnosis of pelvic metastasis from gastric cancer. This case report illustrates that stomach cancer has the potential, although far less than breast, prostate and lung cancers, to metastasize to bone. In addition, it highlights the peculiarity of this bone metastasis which is pelvic, solitary and huge.

## INTRODUCTION

Gastric cancer is the fourth most prevalent malignant tumor worldwide, responsible for approximately 934,000 new diagnoses and 700,349 deaths annually[1]. In contrast, bony skeleton metastases from gastric cancer are uncommon, with an incidence of 13.4% in autopsy specimens[2]. Furthermore, solitary bony metastasis as the first sign of gastric cancer is even rarer[3]. Here, we present an unusual case identified as gastric adenocarcinoma with a single enormous pelvic metastasis as the first clinical manifestation.

## CASE REPORT

**Fig. 1:**
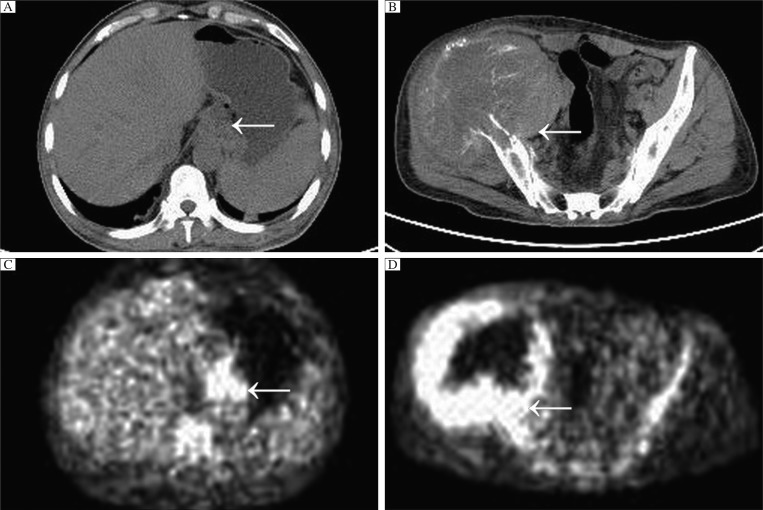
Pelvic metastasis of the cardia adenocarcinoma in a 49-year-old man. Axial unenhanced CT shows a thickened mass (arrow) in the cardia area (A) and an irregular-form soft tissue lump (arrow) in the right pelvis with osteolytic destructions of the right iliac bone (B). PET image exhibits high radioactive uptake (arrow) (C) and demonstrates ring-shaped high radioactive uptake (arrow) of the right pelvic mass (D).

A 49-year-old man was referred to our department in January 2011, with a complaint of pain for the past 5 months and edema for the past 3 months in his right lower limb. Five months earlier, a vague pain occurred in his right hip, then gradually increased and became intolerable, in addition to a novel symptom of edema three months ago. Due to the severe pain and edema, the patient gradually lost the ability to walk and experienced anorexia, insomnia and a 10 kilogram weight loss. One week prior to admission, an outpatient systemic computed tomography (CT) examination had been performed on him. CT findings showed thickening of the local soft tissues of the gastric cardia (***Fig. 1A***), coexisting with osteolytic changes of the right ilium in the periacetabular area, where a soft tissue mass (13.8 cm×12.2 cm×13.5 cm) was located (***Fig. 1B***). The head, thorax and other regions showed normal scanning images. To exclude other potential lesions that were difficult to detect by CT scanning, a positron emission tomography (PET) was then taken. Interestingly, PET found only two abnormally high radioactive uptake areas, in the gastric cardia and right pelvis, which conformed well to CT images (***Fig. 1C*** and ***1D***).

Past history of the patient was unremarkable. The patient occasionally took codeine/paracetamol based analgesics to palliate his pain and discomfort. He was divorced, had one son and worked as an artist. He had a tobacco-consumption history (smoking 10 to 20 cigarettes per day for more than 20 years) and only drank alcohol occasionally. Physical examination noted an approximately 10 cm×10 cm, hard, immovable subcutaneous mass in the right hip and severe lower right extremity edema. No apparent abnormalities of the chest, abdominal, or nervous systems were uncovered. Notably, vascular ultrasonic Doppler of the lower extremities revealed nothing but the anteriorly oppressed right external iliac vein without thrombosis.

Routine laboratory tests displayed minor normocytic anemia (hemoglobin 84 g/L, mean corpuscular volume 90.9fL, mean corpuscular hemoglobin 29 pg), marked hypoproteinemia (albumin 25.2 g/L), and elevated serum alkaline phosphatase (ALP 440.4 IU/L). Tumor markers including carcinoembryonic antigen (CEA) and carbohydrate antigen 199 (CA199) were normal. The remaining parameters, including renal function, serum electrolyte levels, blood coagulation, urine and stool testing, were all within normal limits.

**Fig. 2:**
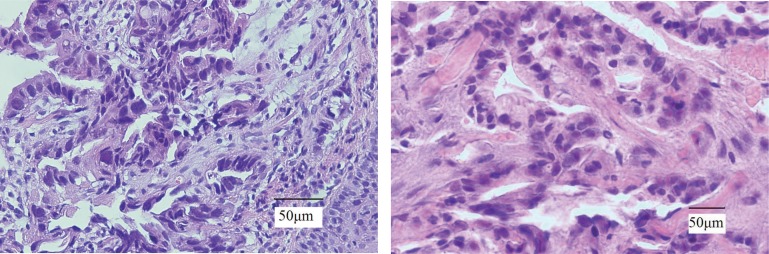
Pathological examination (H&E×400). A: Histology of gastric cardia biopsies reveals squmous epithelium infiltrated with moderately differentiated adenocarcinoma cells, Grade II. B: Metastatic adenocarcinoma cells are evident microscopically in pelvis mass.

In the end, an endoscopic examination of the upper gastrointestinal system was recommended and disclosed a tumoral lesion in the esophago-cardial region, beginning 38 cm away from the incisors and spreading through the cardia of the stomach. Multiple biopsies of the lesion were taken and pathological examination reported moderately-differentiated adenocarcinoma, grade II (***Fig. 2A***). In addition, fine needle aspiration of the pelvic lump was conducted and subsequent pathological investigations revealed metastatic adenocarcinoma (***Fig. 2B***). Taken together, a diagnosis of stage IV cardiac gastric cancer with pelvis metastasis was safely made.

The patient received supportive and symptomatic treatments to reduce cancer-related complications such as pain, anemia, hypoproteinemia, and bone destruction. Transarterial embolization and infusion aimed at the pelvic tumor were subsequently carried out, using therapeutic agents including tegafur (1,000 mg) and cisplatin (60 mg). As expected the pain, edema, anorexia and insomnia were substantially alleviated. Despite choosing not to receive intravenous chemotherapy due to private reasons, the patient survived for three months with a well-improved quality of life, according to the records from follow-up visits.

## DISCUSSION

Bone metastases appear in almost all malignant tumors, with prostate, lung, and breast cancer frequently involved[4]. Indeed, bone ranks third among the most common metastatic sites, just behind the lung and liver[4]. However, osseous pelvic metastasis seems clinically infrequent in stomach cancer. Based on Japanese literature, the incidence of bone metastasis of gastric cancer was 13.4% (33/246) in autopsies[2], far higher than that (1.5%, 33/2242) in resected cases[5].

The mechanisms of bone metastasis in stomach cancer has been widely studied. Lenhert *et al*.[6] hypothesized that it is the rich supply of blood capillaries in gastric mucosa that contribute to the bone metastasis. Another theory proposed by Batson[7] argued that metastasis to the spine may be through the paravertebral venous plexus because intrathoracic or intraabdominal pressure is increased. Additionally, the thoracic duct was regarded as one of the metastatic pathways for gastric cancer to metastasize to the bone[8].

As a general rule, gastric cancer patients are generally characterized with symptoms from primary foci such as epigastric pain, anorexia, vomiting, dysphagia, and weight loss, etc.[9]; those exhibiting symptoms attributable to metastases account for only 5% of all cases[10]. In this case, things were a little more complex. First, the major complaints stemmed from the metastatic pelvic lesion and had little to do with the primary source. In other words, it was the nerve invasion and vein oppression from the pelvic metastasis that directly caused pain and edema in the right lower extremity, not the stomach cancer itself. Second, it was difficult to differentiate the pelvic neoplasm as being metastatic or primary. Although medical history reviews, physical examinations, and routine laboratory tests found a variety of clues highly suggestive of pelvic malignancy, they were not specific to bone metastasis, let alone to that from gastric cancer.

Moreover, bone metastasis from gastric cancer is generally indicative of extensive metastatic disease with preceding or coexisting liver and lung involvement[11]. In this case, however, the right pelvic mass was the sole metastatic site secondary to gastric carcinoma, for CT and PET investigations failed to detect other occult tumoral lesions. Although a solitary pelvic metastasis could be easily mistaken to be a primary osseous tumor, a previous study suggested that a bone tumor in patients beyond 40-year-old should be primarily suspected as a metastatic lesion until proven otherwise[12]. This 49-year-old patient was, theoretically, supposed to have a metastatic rather than primary malignancy, and subsequent pathological examinations did confirm this.

Frequent locations for gastric cancer metastasis are the thoracic and lumbar vertebra[5], while in this case gastric cancer spread specifically to the right pelvis, a comparatively rare site. Pelvic metastases of stomach cancer had been previously reported to occur in 6 out of 2243 patients in a Japanese study[13]. Another article documented a patient diagnosed with gastric adenocarcinoma with multiple osteoblastic metastases to bony structures including the pelvis[14]. Unlike these recorded cases, our patient possessed an extraordinarily large, single pelvic metastasis, measuring 13.8 cm×12.2 cm×13.5 cm, which is very infrequently mentioned in the available literature.

Overall, gastric cancer patients with osseous metastases carry a dismal prognosis with a mean survival time of less than 5 months; 3.5 years has been the longest survival period reported in the literature[14]. Nevertheless, this poor prognosis is superior to that of patients with liver or lung secondaries[11] because most of the bone-related complications (pain, pathological bone fracture, hypercalcemia, and spinal cord compression) are controllable. Accordingly, a multidisciplinary antitumor strategy is advocated for the management of not only bone metastasis, but also for the gastric cancer itself. All effective measures, such as surgical approach, radiotherapy, chemotherapy, transarterial embolization, analgesics, bisphosphonates, and the best supportive treatments are recommended to be cautiously considered and appropriately arranged. If responsive to treatments, even patients with stage IV disease may achieve prolonged survival time and better quality of life.
